# Microbiome composition and central serotonergic activity in patients with depression and type 1 diabetes

**DOI:** 10.1007/s00406-023-01694-8

**Published:** 2023-10-17

**Authors:** Vera Flasbeck, Julia Hirsch, Frank Petrak, Juris J. Meier, Stephan Herpertz, Sören Gatermann, Georg Juckel

**Affiliations:** 1 Department of Psychiatry, LWL-University Hospital, Ruhr University Bochum, Bochum, Germany; 2 Department of Psychosomatic Medicine and Psychotherapy, LWL-University Hospital, Ruhr-University Bochum, Alexandrinenstr.1, 44791 Bochum, Germany; 3 Diabetes Division, Katholisches Klinikum Bochum, St. Josef-Hospital, Ruhr-University Bochum, Bochum, Germany; 4 German National Reference Centre for Multidrug-Resistant Gram-Negative Bacteria, Department of Medical Microbiology, Ruhr-University Bochum, Bochum, Germany

**Keywords:** Microbiome, Serotonin, Tryptophan, Depression, Diabetes, LDAEP

## Abstract

The role of gut–brain axis functioning gains growing attention in research on the pathophysiology of major depressive disorders. Here, especially consequences of altered microbiota composition on tryptophan metabolism resulting in altered serotonergic neurotransmission in the central nervous system (CNS) have reached a central position. Previous research, however, mainly focused on either microbiota and peripheral serotonin levels or central serotonergic neurotransmission. The present study aimed to combine the analysis of microbiota composition and central serotonergic activity using a valid neurophysiological indicator. We recruited 19 adult patients with type 1 diabetes and depression (D + D; 7 males), 19 patients with type 1 diabetes (D-; 7 male), and 20 healthy participants (HC; 7 males). Next to the analysis of fecal microbiota regarding α- and β-diversity, the loudness dependence of auditory evoked potential (LDAEP) was investigated, a non-invasive measurement of central serotonergic activity. High α-diversity was associated with high LDAEP, i.e., low serotonergic activity, in patients with diabetes and additional depression. Furthermore, relative abundances of bacterial families belonging to Bacteroidetes, Proteobacteria and Firmicutes were shown to have an impact on central serotonergic activity. This finding was supported by a tendency indicating an association of central serotonergic activity with the Bacteroidetes–Firmicutes ratio in both patients’ groups. Together, this data suggests that the guts’ microbiota composition might play an important role in regulating the central serotonergic activity in the brain.

## Introduction

Previous research has suggested that dysfunctions of the serotonergic system are causal for the development of psychiatric disorders including depressive disorders [1, 2]. Especially reduced serotonergic availability and neurotransmission in the brain were frequently related to depressive disorders, whereas serotonin (5-hydroxytryptamine, 5-HT) levels were not simply suggested to be reduced in patients with major depressive disorders, instead metabolite levels such as 5-hydroxyindoleacetic acid (5-HIAA) were found to be altered in these patients in the cerebrospinal fluid (CSF [3, 4]). Further evidence was gained by studies using the loudness dependence of auditory evoked potential (LDAEP), which is a non-invasive tool to measure the serotonergic activity in the brain. This method is based on the theory that serotonergic neurotransmission modulates sensory processing in the primary auditory cortex via innervations from the dorsal raphe nuclei [5, 6]. Thus, high levels of activity of serotonergic neurons in the raphe nuclei would reduce the intensity of auditory evoked potentials to increasing auditory stimulation [7]. Moreover, intensity dependence of sensory evoked potentials was also inversely related to 5-HIAA levels in the CSF [8]. Accordingly, a predictive role of LDAEP in responsiveness to treatment with serotonergic antidepressants has been reported in patients with depression [9–11].

Previous research further aimed to investigate whether a dysfunction of the serotonergic system could be related to altered availably of its essential precursor amino acid tryptophan or altered turnover into serotonin. The only way of tryptophan intake is the ingestion via food consumption, whereas especially bananas, peanuts, chocolate, etc., contain high levels of tryptophan. In the gastrointestinal system, tryptophan is metabolized to serotonin, kynurenine or ligands of the aryl hydrocarbon receptor (AhR) [12]. In total, approximately 95% of serotonin is synthesized and stored in the guts, mainly in enterochromaffin cells [13, 14]. In addition, microorganisms in the gut also metabolize tryptophan, and the products mainly affect intestinal AhR activity [12]. There are several species of the gut microbiota, which produce tryptamine from tryptophan directly [15]. Previously, it has been suggested that even if serotonin is synthesized in the gut, it does not contribute to central serotonergic activity, since serotonin itself cannot cross the blood–brain barrier. Therefore, it has been postulated that serotonin availability in the brain depends on the availability of tryptophan, which crosses the barrier via the large amino acid transporter. However, more recently, research proposed instead that peripheral serotonin and other metabolites are able to affect central brain functioning via the vagus nerve, which might serve as an interface [16]. More detailed, serotonin, synthesized by enterochromaffin cells, binds to 5-HT3 receptors on terminals of vagal afferents, which leads to an increased glutamatergic synaptic transmission to second-order neurons of the nucleus tractus solitarius within the brainstem [17, 18].

Taken together, research has suggested that the so called brain–gut microbiome axis exhibits bidirectional communication between brain and guts, affects neurotransmission in general, and is further sensitive to immune and stress responses [19, 20]. Indeed, inflammation and hypothalamic–pituitary–adrenal (HPA) axis activity were found to influence the guts’ microbiota composition [20–22]. Regarding depressive disorders, it could be proposed that processes such as inflammation or stress may induce increased tryptophan metabolism to kynurenine via indoleamine 2,3-dioxygenase (IDO) or tryptophan-2,3-dioxygenase (TDO), and therefore reduced tryptophan availability. This might further lead to lower levels of tryptophan arriving in the brain, which could be metabolized to serotonin to a lower extent [23]. Moreover, altered synthesis of serotonin in the guts could further affect central serotonin levels via vagus nerve projections, which are further known to be involved in psychopathology including depression [24]. Thus, the brain–gut axis could also play an important role for the development of a depression. Regarding the gut microbiota, studies with humans have shown that the gut microbiota impact on anxiety and depression (for review see [23]). In patients with major depressive disorder, altered microbial compositions were found when compared to unaffected control participants [25–27]. More robust evidence for associations of guts microbiota and psychiatric symptoms was gained by animal studies [28]. In any event, the recent findings brought us to the conclusion that the brain and gut should be analyzed simultaneously to assess the pathophysiology of depression. Next to psychiatric disorders, altered gut microbiota has been observed in metabolic disorders including obesity and diabetes [29, 30]. Hence, since psychiatric and metabolic disorders frequently occur together, more effort in disentangling influencing factors and exploration of brain–gut axis functioning would be worthwhile for better understanding of the etiology of depression. Therefore, the present study aimed to investigate the association of microbiota diversity and central serotonergic activity in a sample of patients with type 1 diabetes (D-), patients with type 1 diabetes and depression (D + D), and healthy control participants (HC). We hypothesized to find associations of diversity with central serotonergic activity, especially in patients with additional depressive disorders, due to the proposed altered serotonergic activity and microbiota in these patients.

## Methods and materials

### Participants

Nineteen patients with type 1 diabetes (seven males, age *M* = 42.21, SD = 15.46), nineteen patients with type 1 diabetes and depression (seven males, age *M* = 44.11, SD = 12.03), and twenty healthy participants (seven males, age *M* = 41.85, SD = 14.15) were recruited for the study from the LWL-University Hospital in Bochum and a diabetes focused practice. For demographic characteristics and psychopathology, see Petrak et al. [31]. Participants were included within an age range between 18 and 65 years. Further inclusion criteria were for the patients group type 1 diabetes for longer than 6 months and a treatment with at least 20 insulin units per day. Exclusion criteria were intake of antibiotics within the last 6 months, gastroenteritis, colonic irrigation, or *Clostridium difficile* infections within the last 3 months. Further general exclusion criteria were pregnancy, celiac disease, chronic diarrhea or obstipation, gastroparesis, nephropathy, proliferative retinopathy, macroalbuminuria, and other chronic inflammatory gut diseases and severe somatic disorders. Patients who were using laxatives regularly were excluded. All participants had to be free of comorbid psychiatric disorders and antidepressant medication.

All participants gave full informed written consent. The study was approved by the Ethics Committee of the Medical Faculty of the Ruhr-University Bochum (project number 18-6345) and is in accordance with the Helsinki Declaration.

### Questionnaires

The questionnaires used were described in detail in [31]. In short, participants completed the depression module of the “Patient Health Questionnaire (PHQ)-9 [32, 33], the “Problem Areas in Diabetes” scale (PAID [34, 35]), the 12-item SSCS variant of the “Trier inventory for chronic stress” (TICS [36, 37]).

### Loudness dependence of auditory evoked potentials: EEG recording and analysis of cortical LDAEP

The participants sat in a comfortable armchair in an electrically shielded and sound-attenuated room. Auditory evoked potentials were recorded with 32 passive non-polarizable Ag–AgCl electrodes mounted on an elastic cap in accordance to the 10–20 system by the BrainAmp MR Amplifier and the BrainVision Recorder software (Version 1.20.001; Brain Products GmbH, Gilching, Germany). The electrode configuration contained 29 EEG channels, 1 ground 1 one reference (placed at FCz). We controlled for ocular artefacts by means of the EOG electrode, which was located 1 cm below the left outer canthus. Impedances were kept at 10 kΏ or below. EEG data were filtered using a band-pass filter of 0.531–70 Hz and collected with a sampling rate of 250 Hz. Ten minutes of resting EEG was recorded with eyes closed to exclude the presence of any significant EEG abnormalities. Auditory stimuli were presented binaurally via earphones (Sony Stereo Headphones MDR-1A, Sony^®^ Corporation) and the Presentation^®^ software (Neurobehavioral Systems, Inc. Version 14.9; Berkeley, CA, www.neurobs.com). Pure sinus tones (1000 Hz, 40 ms, 10 ms r/f, ISI 1841–2239 ms, mean 2046 ms) of five different intensities (60, 70, 80, 90, 100 dB sound pressure level) were presented in a pseudorandomized order. A total of 350 sweeps, 70 per intensity, with an epoch length of 800 ms (− 200–600 ms) was evaluated.

Data analysis was carried out using the BrainVision Analyzer 2.0 (Version 2.01.3931; Brain Products GmbH, Gilching, Germany). In detail, re-referencing to the mastoid electrodes was conducted and a notch filter and high- and low-pass filters were applied (low-pass filter 0.5 Hz and high-pass filter 20 Hz). The first five responses to each intensity were also excluded to reduce short-term habituations effects. After segmentation into the loudness levels, the epoch − 200 ms before stimulus onset was used for baseline correction. Epochs with excessive eye or body movements (± 100 μV) in any of the 32 channels were rejected. For each subject, the remaining epochs were averaged separately for the five intensity levels. At least 30 artefact-free sweeps in any of the intensities were required. The N1 amplitude was regarded as the nadir between 50 and 150 ms after the stimulus and P2 as the peak between 100 and 250 ms post-stimulus. The N1/P2 amplitude was then calculated as the difference of peak amplitudes between N1 and P2. The LDAEP of the scalp data (Cz, C3, and C4) was calculated as a median exponential slope of the amplitudes of the single loudness levels [7].

### sLORETA analysis of source LDAEP

For the analysis of source LDAEP values of the primary and secondary auditory cortices, sLORETA [38] was used. Therefore, the re-referencing was conducted to the average of all electrodes and the remaining sweeps were shortened to 300 ms epochs, separately for the five intensity levels. A ROI analysis was performed to investigate the electric neuronal activity as current source density (CSD) in the right and left Heschl’s gyrus (BA41) and for BA42 for the LDAEP of the five intensities between 50 and 250 ms using the eLORETA software [38]. In this study, the BA41-ROI covered a region extended in Talairach space from x: 35 to 55 and − 35 to − 55, y: − 15 to − 40, z: 5–15 and included all voxels of BA41. The BA42-ROI covered the region from x: 55–70 and − 55 to − 65, y: − 10 to − 30, z: 5–20, also including all voxels. The ROI analysis was done with the “ROI-Extractor” tool which averages the CSD values in the specified voxels. The brain model of eLORETA is based on the Montreal Neurological Institute average MRI brain map (MNI 152), while the solution space is limited to the cortical gray matter, comprising 6239 voxels of 5 mm^3^ resolution. The validity of the eLORETA approach as a reliable and effective tool for examining brain activations has been confirmed by several neuroimaging studies using intracranial EEG [39], EEG [40], structural MRI [41], and fMRI (42; 43).

### Microbiota analysis

Stool samples were collected in stool transport tubes (StorAX, Axon Lab AG, Switzerland) and sent within 48 h. The samples were centrifuged at 8000 g for 60 s, and the supernatants were discarded. The pellets’ wet weight was determined and the pellets were frozen at − 70 °C.

#### DNA isolation and detection

The DNA of the gut microbiome was extracted using a commercially available kit (QIAamp Fast DNA Stool Mini Kit, Qiagen, Germany) according to the manufacturer's instructions.

In short, ca 200 mg frozen sample was solved in 1 ml Inhibitex Buffer and continuously vortexed until the stool sample was thoroughly homogenized. The sample was incubated at 95 °C for 5 min and centrifuged for 1 min to remove inhibitors and pellet stool particles. After centrifugation, 400 µl supernatant was added to 30 µl proteinase K and 400 µl buffer AL, and incubated at 70 °C for 10 min. 400 µl ethanol was added, and the sample was loaded to the QIAamp Spin Colum and centrifuged for 1 min at 15,000 g. Then the DNA was washed two times and eluted with 100 µL elution buffer. The Qubit 1×HS dsDNA Assay kit (Invitrogen, ThermoFisher Scientific, Germany) was used for the measurement of DNA concentrations. In addition, we checked for damage and degradation by agarose gel (0.8%) electrophoresis. The samples were probed for bacterial 16S ribosomal DNA (rDNA). For the polymerase chain reaction (PCR), we used the AmpliTaq Gold Master-Mix (Applied Biosystem, ThermoFisher Scientific, Germany) and the following primers: 16s_27F 3′-AGAGTTTGATCMTGGCTCAG-5′ and 16s_926R 3′-CCGTCAATTCCTTTRAGTTT-5′. Illumina Sequencing was performed by a commercial vendor (Novogene Europe, Cambridge, UK). In short, sequencing libraries were generated using NEBNext Ultra DNA Library Pre^®^Kit for Illumina, according to the manufacturer’s instructions, and index codes were added. The library quality was assessed on a Qubit@ 2.0 Fluorometer (Thermo Scientific) and Agilent Bioanalyzer 2100 system. Finally, the library was sequenced on an Illumina platform and 250 base pair (bp) paired-end reads were generated. One sample from a healthy participant was not analyzed successfully.

#### Bioinformatic analyses

The resulting sequences were trimmed and paired-end reads were merged using FLASH (V1.2.7, http://ccb.jhu.edu/software/FLASH/). After quality control, sequences were de-replicated and chimeras were removed (UCHIME http://drive5.com/uchime/uchime_download.html using the GOLD database). Operating taxon units (OTUs) were clustered at 97% similarity using USEARCH. The taxonomic annotations were done using the RDP classifier (Version 2.2, http://sourceforge.net/projects/rdp-classifier/) with the GreenGenes database (http://greengenes.lbl.gov/cgi-bin/nph-index.cgi) for reference. The software R (V 3.6.3) [42] and the packages vegan [43] phyloseq [44] were used for downstream analysis. Data were standardized to contain the same number of sequences per sample using the sample with the lowest number as the reference. For alpha diversity, Chao1, ACE, and the Shannon index were computed. Beta-diversity was analyzed with Bray–Curtis distances.

### Statistical analysis

Statistical analyses were carried out using IBM SPSS Statistics for Windows, version 26 (IBM Corp., Armonk, NY) and the Software R (V 3.6.3) [42]. The LDAEP results were compared between groups (D-, D + D, HC) by MANOVA for C3, C4, Cz, BA41-L, BA41-R; BA42-L, BA42-R. For the α-diversity, ACE, Chao1, and Shannon index and the number of species were analyzed. Pearson correlation coefficients were calculated to assess correlations between LDAEP and psychometric data and α-diversity. Differences in microbiota composition, i.e., microbial abundances, between participants with low and high central serotonergic activity, i.e., LDAEP, were calculated by Wilcoxon signed-rank test with groups build based on median splits (< = median; > median). Pearson correlations of C3, Cz, C4 and BA41 and BA42 (L/R; left and right, respectively) with the occurrence of bacteria families and the Bacteroidetes/Firmicutes ratio were calculated for the whole sample. Correlations for the ratio were additionally calculated within subgroups. Only taxa with a median count > 10 in both groups were reported. For comparison of Bray–Curtis distances, the permutational analysis of variance (PERMOVA) implementation of the vegan package (adonis function) was used. For identification of significant differences between the low and high LDAEP groups, the TukeyHSD function was utilized. A *p* value < 0.05 was considered significant.

## Results

### LDAEP and correlations with psychometric data

Comparisons of LDAEP values (see Table 1 for descriptive statistics) between groups using MANOVA did not reveal a significant main effect of group (*F*(14, 98) = 0.562, *p* = 0.888, partial *η*^2^ = 0.074, Wilk’s *Λ* = 0.857).

**Table 1:** Descriptive statistics of LDAEP values in patients with type 1 diabetes (D-), patients with type 1 diabetes and depression (D + D), and healthy controls (HC)

Electrode/region	Patients with D-	Patients with D + D	HC
C3	0.145 (0.102)	0.194 (0.115)	0.160 (0.112)
C4	0.154 (0.106)	0.180 (0.111)	0.157 (0.126)
Cz	0.218 (0.106)	0.263 (0.154)	0.221 (0.166)
BA41-R	0.152 (0.127)	0.189 (0.149)	0.229 (0.144)
BA41-L	0.189 (0.178)	0.196 (0.197)	0.231 (0.124)
BA42-R	0.160 (0.158)	0.191 (0.100)	0.219 (0.158)
BA42-L	0.218 (0.244)	0.223 (0.250)	0.248 (0.205)

Mean values and standard deviations are shown

Interestingly, when correlations of LDAEP with psychometric data were calculated for the whole sample, PAID was found to correlate with LDAEP over C3 (*r* = 0.511, *p* = 0.001), C4 (*r* = 0.353, *p* = 0.030), and Cz (*r* = 0.388, *p* = 0.016). Analyses of the groups separately revealed again a correlation of PAID with LDAEP over C3 (*r* = 0.521, *p* = 0.022) in the D + D group. No correlation emerged in the other groups and no correlations occurred with PHQ-9 or TICS. In other words, the results showed that low serotonergic activity was related to increased diabetes distress in patients with additional depressive symptoms.

### Microbiota

#### α-diversity

**Fig. 1:**
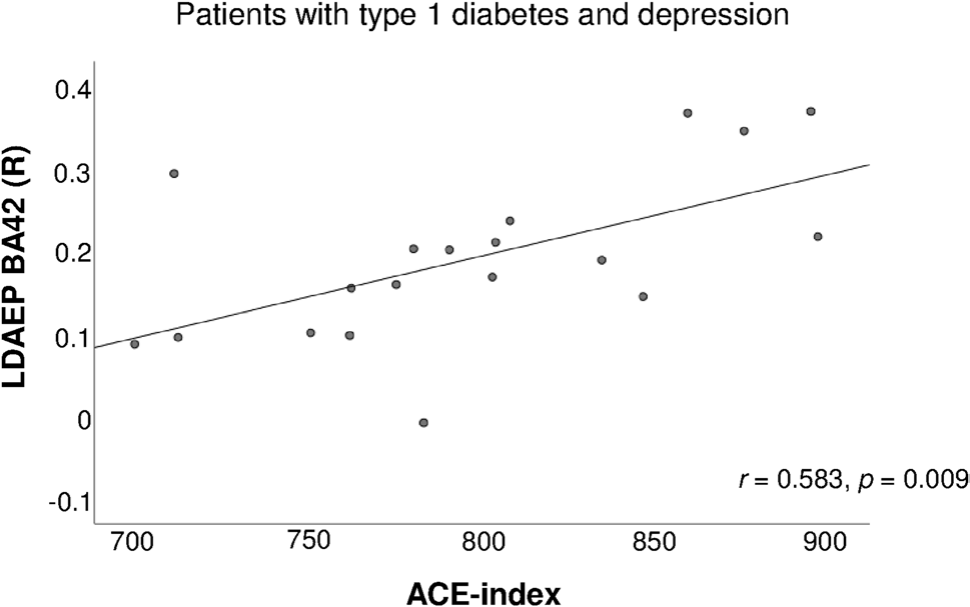
Scatter plot showing the association of the LDAEP over BA42 (R) and the ACE index in patients with type 1 diabetes and depression

Here, we focused on correlations between α-diversity and LDAEP. In the D + D group, α-diversity indices correlated with LDAEP over BA42-R (Shannon index: *r* = 0.514, *p* = 0.025; No. species: *r* = 0.616, *p* = 0.005; Chao1: *r* = 0.622, *p* = 0.005; ACE: *r* = 0.583, *p* = 0.009; see Fig. 1). This indicates that increased diversity was associated with high LDAEP, i.e., low serotonergic activity in patients with diabetes and additional depression.

#### Relative abundances

The LDAEP over C3, C4, Cz and BA41 and BA42 on the left and right sides correlated with the occurrence of various bacterial families (Table 2) belonging to the phyla Actinobacteria, Bacteroidetes, Proteobacteria, Firmicutes, Verrucomicrobia, Spirochaetes, and Lentisphaerae.

**Table 2:** Results of correlation analyses between abundances of bacterial families and LDAEP results of distinct regions and electrodes

Electrode region	Correlations family (test statistics: *p*)	Phylum
C3	Intrasporangiaceae (0.041)	Actinobacteria
	Rickenellaceae (0.043)	Bacteroidetes
C4	Idiomarinaceae (0.027)	Proteobacteria
	Pasteurellaceae (0.029)	Proteobacteria
	Rickenellaceae (0.020)	Bacteroidetes
Cz	Idiomarinaceae (0.033)	Proteobacteria
	Family XI (0.012)	Firmicutes
	Lactobacillaceae (0.047)	Firmicutes
	Pasteurellaceae (0.013)	Proteobacteria
BA41-R	Desulfovibrionaceae (0.042)	Proteobacteria
	Enterococcaceae (0.002)	Firmicutes
	Verrucomicrobiaceae (< 0.001)	Verrucomicrobia
BA41-L	Brevinemataceae (0.029)	Spirochaetes
BA42-R	Campylobacteraceae (0.029)	Proteobacteria
	Clostridiales vadin BB60 (< 0.001)	Firmicutes
	Defluviitaleaceae (0.012)	Firmicutes
	Desulfovibrionaceae (< 0.001)	Proteobacteria
	Enterococcaceae (< 0.001)	Firmicutes
	Lachnospiraceae (0.046)	Firmicutes
	Victivallaceae (< 0.001)	Lentisphaerae
BA42-L	Succinivibrionaceae (0.032)	Proteobacteria

Only significant results are shown. The right column indicates the phylum the families are belonging to

When conducting median splits for the LDAEP values, the Wilcoxon test revealed differences between the low LDAEP and the high LDAEP group regarding the occurrence of distinct bacterial families from Bacteroidetes, Proteobacteria, Firmicutes, and Verrucomicrobia phyla (Table 3).

**Table 3:** Results of group comparisons for low LDAEP and high LDAEP groups

Electrode region	Wilcoxon signed-rank test family (test statistics: *W; p*)	Phylum
C3	Rickenellaceae (*W* = 557; *p* = 0.015)	Bacteroidetes
	Desulfovibrionaceae (*W* = 532; *p* = 0.045)	Proteobacteria
	Oxalobacteraceae (*W* = 585; *p* = 0.011)	Proteobacteria
C4	Defluviitalaceae (*W* = 266; *p* = 0.025)	Firmicutes
Cz	Bacteroidales S24-7 (*W* = 551; *p* = 0.020)	Bacteroidetes
	Clostridiaceae_1 (*W* = 272; *p* = 0.032)	Firmicutes
	Defluviitalaceae (*W* = 265; *p* = 0.024)	Firmicutes
BA41_R	Verrucomicrobiaceae (*W* = 190; *p* < 0.001)	Verrucomicrobia
BA41_L	Peptostreptococcaceae (*W* = 280; *p* = 0.045)	Firmicutes
	Streptococcaceae (*W* = 267; *p* = 0.026)	Firmicutes
	Lactobacillaceae (*W* = 267; *p* = 0.026)	Firmicutes
BA42_R	Prevotellaceae (*W* = 566; *p* = 0.010)	Bacteroidetes
	Alcaligenaceae (*W* = 281; *p* = 0.046)	Proteobacteria
	Verrucomicrobiaceae (*W* = 209; *p* = 0.001)	Verrucomicrobia
BA42_L	Peptococcaceae (*W* = 551; *p* = 0.020)	Firmicutes

Only significant differences between groups for the denoted families are shown

**Fig. 2:**
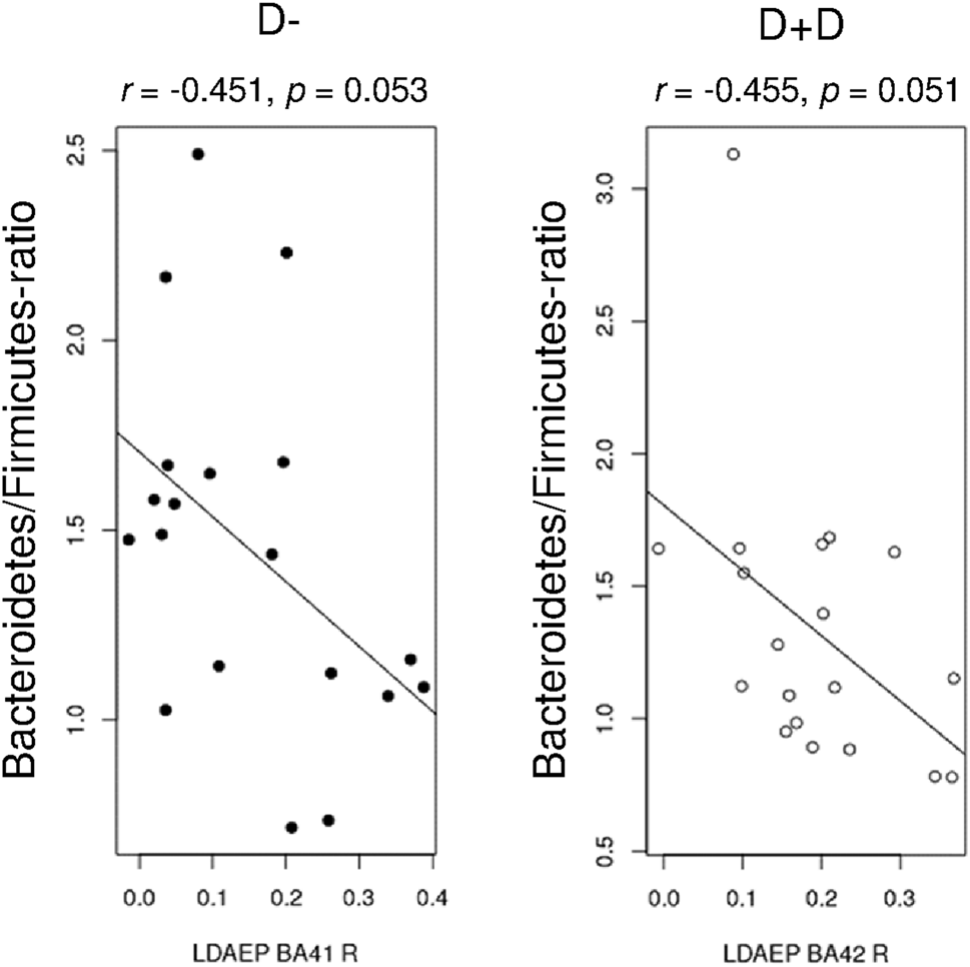
Correlations between the Bacteroides/Firmicutes ratio and LDAEP measured in BA41-R for patients with diabetes (filled circles: left) und LDAEP measured in BA42-R for D + D patients (D-D, open circles; right)

When calculating the Bacteroidetes/Firmicutes ratio, correlations were found between the ratio and LDAEP in the patients’ groups (D- group: correlation of ratio with LDAEP in right BA41: *r* = − 0.451; *p* = 0.053; D + D group: correlation of ratio with LDAEP in right BA42: *r* = − 0.455, *p* = 0.051; see Fig. 2).

#### β-diversity

**Fig. 3:**
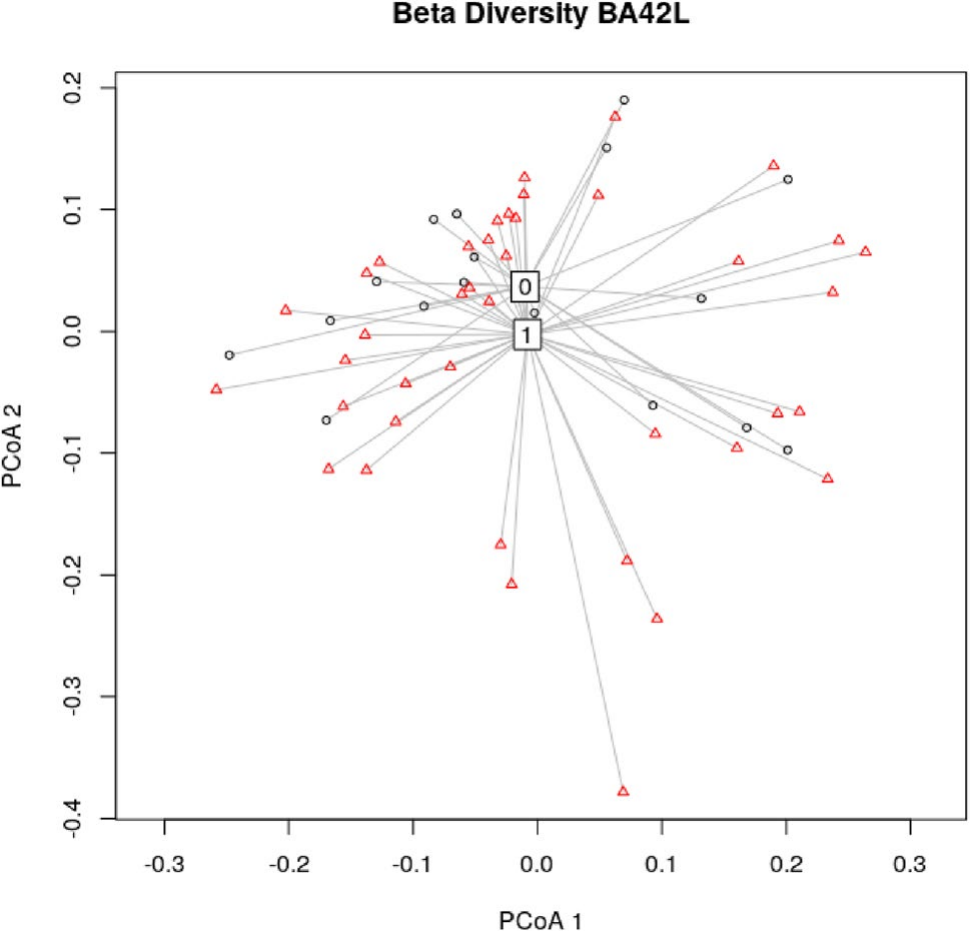
Depiction of β-diversity for groups with high (i.e., low LDAEP = 0) and low (i.e., high LDAEP = 1) central serotonergic activity

A tendency for a difference in β-diversity between groups (low vs. high LDAEP group) was found for BA42-L (*F*(1, 55) = 3.844, *p* = 0.055; Fig. 3).

For differences between groups in α-and β-diversity, please see Petrak et al. [31].

## Discussion

The present study focused on the association of microbiota composition and serotonergic activity in patients with type 1 diabetes and depression (D + D), patients with type 1 diabetes (D-), and healthy controls (HC). We expected to detect correlations between α-diversity, β-diversity, and central serotonergic activity, especially in the group of patients with a depressive disorder. Here, increased diabetes distress was related to low serotonergic activity, in all patients and in patients with diabetes and additional depressive symptoms alone. We would like to note that the PAID questionnaire, which investigates diabetes distress, strongly correlated with depressive symptoms [45], and therefore the PAID results should not be considered as a measure of pure diabetes-specific distress. Previous research on LDAEP in patients with depression brought inconsistent results regarding altered serotonergic activity in patients with depression [9, 46, 47]. In contrast, a predictive role of LDAEP for SSRI-treatment response has been shown frequently [9–11, 47] as well as relationships between clinical characteristics and LDAEP [48–50]. Here, we also did not detect differences between groups. As mentioned above, increased diabetes distress correlated with high LDAEP, i.e., low serotonergic activity, which is consistent with previous studies. LDAEP was further correlated with α-diversity in the D + D group, whereby increased diversity, measured by various α-diversity indices, was associated with high LDAEP over BA 42 (right), i.e., low serotonergic activity. This finding would implicate that high α-diversity would be unfavorable for humans, which sounds counterintuitive. However, this suggestion is in line with the results from our previous publication, which shows lower diversity in the control group compared to both patients groups, which again supports the impression of higher diversity being “more pathological” (for details see [31]). A higher Shannon index was also reported by another group for patients with depression compared to control groups [25]. In a study of Zheng and colleagues, no significant differences in α-diversity between patients with depression and a healthy control group were observed [27] and another publication demonstrated lower α-diversity indices in patients [51]. Thus, previous research also brought inconsistent results regarding α-diversity and depression. Accordingly, two recent review articles further concluded that no altered α-diversity has reliably been found in patients with MDD [52, 53]. When focusing on the association between α-diversity and serotonin metabolism, less research was conducted to date. A group investigated the effect of *Lactobacillus plantarum* DR7 administration for 12 weeks in stressed adults and found higher α-diversity in the *Lactobacillus plantarum* DR7 group compared to a placebo group [54], as well as reduced psychopathology, plasma cortisol and pro-inflammatory cytokine levels, and increased anti-inflammatory cytokine levels. Most interestingly, in the experimental group, the serotonin pathway was enhanced, which was suggested to be based on lower expressions of tyrosine hydroxylase, IDO and TDO, and higher tryptophan hydroxylase-2 and 5-hydroxytryptamine receptor-6 expression [55]. The authors further reported that especially the abundances of Bacteroidia and Bacteroidales correlated with tryptophan hydroxylase-II [54]. Accordingly, especially for depression, a therapeutic effect of probiotics, which enhances the amount of beneficial bacteria in the gut, on depressive symptoms has been suggested [52, 56].

In our study, we found correlations of LDAEP with the occurrence of bacterial families belonging mainly to Bacteroidetes, Proteobacteria, and Firmicutes. These findings were also confirmed by comparisons of the abundances between the low and high LDAEP groups, as defined by median splitting. The bacterial phyla and classes differ in their characteristics in terms of metabolisms, as for example anaerobe metabolism is occurring in the class Clostridia. In contrast, members of the class Bacteroidea and phylum Bacteroidetes are Gram-negative, aerobic or anaerobic bacteria. Thus, an altered composition of the guts microbiota may affect the metabolism. The Bacteroidetes–Firmicutes ratios were further related to central serotonergic activity in the patients’ groups on trend level, which again supports the association of microbiota composition with central serotonergic activity. The mechanisms behind the association of bacterial abundances and central serotonergic activity are still a matter of discussion. One possible pathway is the impact of short-chain fatty acids, which are bacterial metabolites, on the serotonergic system via regulation of inflammation. The short-chain fatty acids acetate, butyrate, and propionate interact with the immune system [57]. For instance, the amount of butyrate is suggested to correlate with the numbers of regulatory T cells, whereas activation of these cells inhibits histone deacetylases (HDAC) [58]. Butyrate further regulates intestinal macrophage functioning by inhibition of the production of inflammatory cytokines [59]. Especially Clostridia were proposed to manipulate regulatory T cells [60]. Since inflammatory cytokines and processes are suggested to impact on tryptophan metabolism, resulting in increased metabolism by IDO, lower levels of tryptophan may be available for turnover into serotonin in the brain [61, 62]. Thus, the microbiota composition, together with dietary habits, may affect the central serotonergic system in the brain.

Regarding the role of bacterial composition in depression, a previous study reported an increase in the relative abundances of Actinobacteria in patients with depression, whereas the abundance of Bacteroidetes was decreased. The relative abundance of Firmicutes was also deviating from control participants. When the fecal microbiota of these patients was transplanted into germ-free mice, the animals showed depression-like behaviors due to altered metabolism, which was not visible after transplantation of “healthy microbiota” [27]. Jiang et al. found higher abundances of Bacteroidetes and Proteobacteria and lower proportion of Firmicutes in patients with depression [25]. Recently, a study by Chen et al. reported significant differences regarding the abundances of Bacteroidetes, Proteobacteria, Firmicutes, and Actinobacteria between patients with depression and healthy participants using comparative metaproteomics analysis [63]. Data of our study showed specific higher abundance of Megasphaera in patients with diabetes and depression when compared to D− and HC groups [31]. Together, our study and previous research suggest that the proportion of bacteria abundances may play a role in depression and in serotonergic system functioning. This is further supported by the difference in β-diversity between the low and high LDAEP group found in our study, although reaching only trend level, and psychopharmacological studies that have shown that fluoxetine, a selective serotonin reuptake inhibitor (SSRI), has an antimicrobial activity against distinct bacteria involved in inflammation processes in the guts [61]. However, future research may clarify the mechanisms behind the interaction of the guts microbiota, inflammatory processes, and the serotonergic system in the brain.

### Limitations

A limitation of the present study is the inclusion of patients with diabetes and additional depression, but a lack of patients with a depressive disorder without diabetes. Therefore, the study precludes any conclusions regarding the role of the microbiome in depressive disorders alone. As previously shown, Bacteroidetes were dominating in preclinical patients with type 1 diabetes, whereas low abundance of butyrate-producing bacteria was further reported [64, 65]. Here, the two disorders presumably may have affected each other. Moreover, the sample sizes are relatively small, wherefore replication in another larger, unrelated sample would be important. For further understanding of the mechanisms of gut–brain communication, measurement of serotonin levels in the blood and feces would be an interesting target, which was not conducted in the present study.

## Conclusion

This is the first study on the interaction of the microbiota and the central serotonergic system that could demonstrate an interaction between the two systems. We found an association of central serotonergic activity with α-diversity and self-reported diabetes distress. In addition, bacterial families belonging predominantly to the phyla Bacteroidetes, Proteobacteria, and Firmicutes were shown to be related to central serotonergic activity. We, therefore, concluded that the guts’ microbiota composition and the balance of phyla may play a crucial role in regulating the central serotonergic activity in the brain.

## Data Availability

Data are available on request.

## References

[1] Meltzer HY (1990). Role of serotonin in depression. Ann N Y Acad Sci.

[2] Owens MJ, Nemeroff CB (1994). Role of serotonin in the pathophysiology of depression: focus on the serotonin transporter. Clin Chem.

[3] Åsberg M, Bertilsson L, Saletu B, Berner P, Hollister L (1979). Serotonin in Depressive Illness—Studies of CSF 5-HIAA. Neuro-Psychopharmacology.

[4] Hou C, Jia F, Liu Y, Li L (2006). CSF serotonin, 5-hydroxyindolacetic acid and neuropeptide Y levels in severe major depressive disorder. Brain Res.

[5] Jacobs BL, Azmitia EC (1992). Structure and function of the brain serotonin system. Physiol Rev.

[6] Lewis DA, Campbell MJ, Foote SL, Morrison J (1986). The monoaminergic innervation of primate neocortex. Hum Neurobiol.

[7] Hegerl U, Juckel G (1993). Intensity dependence of auditory evoked potentials as an indicator of central serotonergic neurotransmission: a new hypothesis. Biol Psychiat.

[8] von Knorring L, Perris C (1981). Biochemistry of the augmenting-reducing response in visual evoked potentials. Neuropsychobiology.

[9] Gallinat J, Bottlender R, Juckel G, Munke-Puchner A, Stotz G, Kuss H-J (2000). The loudness dependency of the auditory evoked N1/P2-component as a predictor of the acute SSRI response in depression. Psychopharmacology.

[10] Juckel G, Pogarell O, Augustin H, Mulert C, Müller-Siecheneder F, Frodl T (2007). Differential prediction of first clinical response to serotonergic and noradrenergic antidepressants using the loudness dependence of auditory evoked potentials in patients with major depressive disorder. J Clin Psychiatry.

[11] Park Y-M, Lee S-H, Park EJ (2012). Usefulness of LDAEP to predict tolerability to SSRIs in major depressive disorder: a case report. Psychiatry Investig.

[12] Agus A, Planchais J, Sokol H (2018). Gut microbiota regulation of tryptophan metabolism in health and disease. Cell Host Microbe.

[13] Richard DM, Dawes MA, Mathias CW, Acheson A, Hill-Kapturczak N, Dougherty DM (2009). L-Tryptophan: basic metabolic functions, behavioral research and therapeutic indications. Int JTryptophanRes.

[14] Sanger GJ (2008). 5-Hydroxytryptamine and the gastrointestinal tract: where next?. Trends Pharmacol Sci.

[15] Williams BB, van Benschoten AH, Cimermancic P, Donia MS, Zimmermann M, Taketani M (2014). Discovery and characterization of gut microbiota decarboxylases that can produce the neurotransmitter tryptamine. Cell Host Microbe.

[16] Bonaz B, Bazin T, Pellissier S (2018). The Vagus nerve at the interface of the microbiota-gut-brain axis. Front Neurosci.

[17] Browning KN (2015). Role of central vagal 5-HT3 receptors in gastrointestinal physiology and pathophysiology. Front Neurosci.

[18] Breit S, Kupferberg A, Rogler G, Hasler G (2018). Vagus nerve as modulator of the brain-gut axis in psychiatric and inflammatory disorders. Front Psych.

[19] Clarke G, Grenham S, Scully P, Fitzgerald P, Moloney RD, Shanahan F (2013). The microbiome-gut-brain axis during early life regulates the hippocampal serotonergic system in a sex-dependent manner. Mol Psychiatry.

[20] O’Mahony SM, Clarke G, Borre YE, Dinan TG, Cryan JF (2015). Serotonin, tryptophan metabolism and the brain-gut-microbiome axis. Special Issue: Serotonin.

[21] Collins SM, Surette M, Bercik P (2012). The interplay between the intestinal microbiota and the brain. Nat Rev Microbiol.

[22] Cryan JF, Dinan TG (2012). Mind-altering microorganisms: the impact of the gut microbiota on brain and behaviour. Nat Rev Neurosci.

[23] Kennedy PJ, Cryan JF, Dinan TG, Clarke G (2017). Kynurenine pathway metabolism and the microbiota-gut-brain axis. The Kynurenine Pathway in Health and Disease.

[24] Tan C, Yan Q, Ma Y, Fang J, Yang Y (2022). Recognizing the role of the vagus nerve in depression from microbiota-gut brain axis. Front Neurol.

[25] Jiang H, Ling Z, Zhang Y, Mao H, Ma Z, Yin Y (2015). Altered fecal microbiota composition in patients with major depressive disorder. Brain Behav Immun.

[26] Kelly JR, Borre Y, O' Brien C, Patterson E, El Aidy S, Deane J, (2016). Transferring the blues: depression-associated gut microbiota induces neurobehavioural changes in the rat. J Psychiatr Res.

[27] Zheng P, Zeng B, Zhou C, Liu M, Fang Z, Xu X (2016). Gut microbiome remodeling induces depressive-like behaviors through a pathway mediated by the host’s metabolism. Mol Psychiatry.

[28] Settanni CR, Ianiro G, Bibbò S, Cammarota G, Gasbarrini A (2021). Gut microbiota alteration and modulation in psychiatric disorders: current evidence on fecal microbiota transplantation. Prog Neuropsychopharmacol Biol Psychiatry.

[29] Everard A, Cani PD (2013). Diabetes, obesity and gut microbiota. The Gut Microbiome.

[30] Musso G, Gambino R, Cassader M (2011). Interactions between gut microbiota and host metabolism predisposing to obesity and diabetes. Annu Rev Med.

[31] Petrak F, Herpertz S, Hirsch J, Röhrig B, Donati-Hirsch I, Juckel G (2022). Gut microbiota differs in composition between adults with type 1 diabetes with or without depression and healthy control participants: a case-control study. BMC Microbiol.

[32] Kroenke K, Spitzer RL, Williams JBW (2001). The PHQ-9. J Gen Intern Med.

[33] Kroenke K, Spitzer RL (2002). The PHQ-9: a new depression diagnostic and severity measure. Psychiatr Ann.

[34] Schmitt A, Reimer A, Kulzer B, Haak T, Ehrmann D, Hermanns N (2016). How to assess diabetes distress: comparison of the problem areas in diabetes scale (PAID) and the diabetes distress scale (DDS). Diabet Med.

[35] Welch GW, Jacobson AM, Polonsky WH (1997). The problem areas in diabetes scale: an evaluation of its clinical utility. Diabetes Care.

[36] Petrowski K, Kliem S, Albani C, Hinz A, Brähler E (2019). Norm values and psychometric properties of the short version of the trier inventory for chronic stress (TICS) in a representative German sample. PLoS ONE.

[37] Petrowski K, Paul S, Albani C, Brähler E (2012). Factor structure and psychometric properties of the trier inventory for chronic stress (TICS) in a representative german sample. BMC Med Res Methodol.

[38] Pascual-Marqui RD (2002). Standardized low-resolution brain electromagnetic tomography (sLORETA): technical details. Methods Find Exp Clin Pharmacol.

[39] Zumsteg D, Friedman A, Wieser HG, Wennberg RA (2006). Propagation of interictal discharges in temporal lobe epilepsy: correlation of spatiotemporal mapping with intracranial foramen ovale electrode recordings. Clin Neurophysiol.

[40] Leicht G, Kirsch V, Giegling I, Karch S, Hantschk I, Möller H-J (2010). Reduced early auditory evoked gamma-band response in patients with schizophrenia. Synaptic Plast Deficits Schizophr.

[41] Worrell GA, Lagerlund TD, Sharbrough FW, Brinkmann BH, Busacker NE, Cicora KM (2000). Localization of the epileptic focus by low-resolution electromagnetic tomography in patients with a lesion demonstrated by MRI. Brain Topogr.

[42] R Core Team (2020) R: a language and environment for statistical computing*.* Vinna, Austria: R Foundation for Statistical Computing

[43] Oksanen J, Blanchet FG, Friendly M, Kindt R, Legendre P, McGlinn D et al. (2019) vegan: community ecology package. R package version 2, 5–6

[44] McMurdie PJ, Holmes S (2013). phyloseq: an R package for reproducible interactive analysis and graphics of microbiome census data. PLoS ONE.

[45] Stanulewicz N, Mansell P, Cooke D, Hopkins D, Speight J, Blake H (2019). PAID-11: a brief measure of diabetes distress validated in adults with type 1 diabetes. Diabetes Res Clin Pract.

[46] Graßnickel V, Illes F, Juckel G, Uhl I (2015). Loudness dependence of auditory evoked potentials (LDAEP) in clinical monitoring of suicidal patients with major depression in comparison with non-suicidal depressed patients and healthy volunteers: a follow-up-study. J Affect Disord.

[47] O'Neill BV, Croft RJ, Nathan PJ (2008). The loudness dependence of the auditory evoked potential (LDAEP) as an in vivo biomarker of central serotonergic function in humans: rationale, evaluation and review of findings. Hum Psychopharmacol Clin Exp.

[48] Jaworska N, Blier P, Fusee W, Knott V (2012). Scalp- and sLORETA-derived loudness dependence of auditory evoked potentials (LDAEPs) in unmedicated depressed males and females and healthy controls. Clin Neurophysiol : Off J Int Fed Clin Neurophysiol.

[49] Mulert C, Juckel G, Augustin H, Hegerl U (2002). Comparison between the analysis of the loudness dependency of the auditory N1/P2 component with LORETA and dipole source analysis in the prediction of treatment response to the selective serotonin reuptake inhibitor citalopram in major depression. Clin Neurophysiol.

[50] Wyss C, Hitz K, Hengartner MP, Theodoridou A, Obermann C, Uhl I (2013). The loudness dependence of auditory evoked potentials (LDAEP) as an indicator of serotonergic dysfunction in patients with predominant schizophrenic negative symptoms. PLoS ONE.

[51] Huang Y, Shi X, Li Z, Shen Y, Shi X, Wang L (2018). Possible association of Firmicutes in the gut microbiota of patients with major depressive disorder. Neuropsychiatr Dis Treat.

[52] Alli SR, Gorbovskaya I, Liu JCW, Kolla NJ, Brown L, Müller DJ (2022). The gut microbiome in depression and potential benefit of prebiotics, probiotics and synbiotics: a systematic review of clinical trials and observational studies. Int J Mol Sci.

[53] Nikolova VL, Hall MRB, Hall LJ, Cleare AJ, Stone JM, Young AH (2021). Perturbations in gut microbiota composition in psychiatric disorders: a review and meta-analysis. JAMA Psychiat.

[54] Liu G, Chong H-X, Chung FY-L, Li Y, Liong M-T (2020). Lactobacillus plantarum DR7 modulated bowel movement and gut microbiota associated with dopamine and serotonin pathways in stressed adults. Int J Mol Sci.

[55] Chong HX, Yusoff NAA, Hor Y-Y, Lew L-C, Jaafar MH, Choi S-B (2019). Lactobacillus plantarum DR7 alleviates stress and anxiety in adults: a randomised, double-blind, placebo-controlled study. Benef Microbes.

[56] Mörkl S, Butler MI, Holl A, Cryan JF, Dinan TG (2020). Probiotics and the microbiota-gut-brain axis: focus on psychiatry. Curr Nutr Rep.

[57] Tilg H, Moschen AR (2015). Food, immunity, and the microbiome. Gastroenterology.

[58] Furusawa Y, Obata Y, Fukuda S, Endo TA, Nakato G, Takahashi D (2013). Commensal microbe-derived butyrate induces the differentiation of colonic regulatory T cells. Nature.

[59] Chang PV, Hao L, Offermanns S, Medzhitov R (2014). The microbial metabolite butyrate regulates intestinal macrophage function via histone deacetylase inhibition. Proc Natl Acad Sci USA.

[60] Atarashi K, Tanoue T, Shima T, Imaoka A, Kuwahara T, Momose Y (2011). Induction of colonic regulatory T cells by indigenous clostridium species. Science (New York, N.Y.).

[61] Waclawiková B, El Aidy S (2018). Role of microbiota and tryptophan metabolites in the remote effect of intestinal inflammation on brain and depression. Pharmaceuticals (Basel, Switzerland).

[62] Yeung AWS, Terentis AC, King NJC, Thomas SR (2015). Role of indoleamine 2,3-dioxygenase in health and disease. Clin Sci (London, England :1979).

[63] Chen Z, Li J, Gui S, Zhou C, Chen J, Yang C (2018). Comparative metaproteomics analysis shows altered fecal microbiota signatures in patients with major depressive disorder. NeuroReport.

[64] Knip M, Siljander H (2016). The role of the intestinal microbiota in type 1 diabetes mellitus. Nat Rev Endocrinol.

[65] Vaarala O (2013). Human intestinal microbiota and type 1 diabetes. Curr DiabRep.

